# Geometry Constrained *N*-(5,6,7-Trihydroquinolin-8-ylidene)arylaminopalladium Dichloride Complexes: Catalytic Behavior toward Methyl Acrylate (MA), Methyl Acrylate-*co*-Norbornene (MA-*co*-NB) Polymerization and Heck Coupling

**DOI:** 10.3390/molecules21121686

**Published:** 2016-12-07

**Authors:** Yanning Zeng, Qaiser Mahmood, Tongling Liang, Wen-Hua Sun

**Affiliations:** 1 Key Laboratory of Engineering Plastics, Beijing National Laboratory for Molecular Sciences, Institute of Chemistry, Chinese Academy of Sciences, Beijing 100190, China; mqaiser@iccas.ac.cn (Q.M.); ltl@iccas.ac.cn (T.L.); 2 State Key Laboratory for Oxo Synthesis and Selective Oxidation, Lanzhou Institute of Chemical Physics, Chinese Academy of Sciences, Lanzhou 730000, China; 3 International School, University of Chinese Academy of Sciences, Beijing 100049, China

**Keywords:** constrained ligand framework, palladium complexes, homopolymerization of MA, MA/NB copolymerization, Heck coupling

## Abstract

A new pair of plladium complexes (**Pd4** and **Pd5**) ligated with constrained *N*-(5,6,7-trihydroquinolin-8-ylidene)arylamine ligands have been prepared and well characterized by ^1^H-, ^13^C-NMR and FTIR spectroscopies as well as elemental analysis. The molecular structure of **Pd4** and **Pd5** in solid state have also been determined by X-ray diffraction, showing slightly distorted square planar geometry around the palladium metal center. All complexes **Pd1**–**Pd5** are revealed highly efficient catalyst in methyl acrylate (MA) polymerization as well as methyl acrylate/norbornene (MA/NB) copolymerization. In the case of MA polymerization, as high as 98.4% conversion with high molecular weight up to 6282 kg·mol^−1^ was achieved. Likewise, **Pd3** complex has good capability to incorporate about 18% NB content into MA polymer chains. Furthermore, low catalyst loadings (0.002 mol %) of **Pd4** or **Pd5** are able to efficiently mediate the coupling of haloarenes with styrene affording up to 98% conversion.

## 1. Introduction

The abundant chemistry of palladium-based complex catalysts has attracted considerable attention, particularly in the field of C–C bond formation, C–H functionalization, hydrocarbon oxidation, as well as radical insertion and addition reaction in polymerization, indicating that palladium is one of the most catalytically versatile transition metals [1,2,3].

The Mizoroki–Heck reaction [4,5,6,7,8,9,10,11] using palladium catalyst has been extensively utilized in the synthesis of natural products [12], pharmaceuticals, bioactive compounds [13], fine chemical substances [14], as well as polymeric materials [15]. Besides mild reaction conditions, Mizoroki–Heck reactions also tolerate various functional groups including amino [16], hydroxyl [17], aldehyde [18], ketone [19], carboxylic acid [20], ester [21], cyano [22], and nitro groups [23] within both the aryl or vinyl halides and alkenes. The extensive use have driven the price of palladium higher and higher, making it is necessary to enhance the catalytic efficiency by designing simpler and general palladium catalyst systems which can work under accessible reaction conditions.

On the other hand, commercial copolymerization processes plays a key role in the production of new materials, which is indispensable in all fields of human life [24,25,26,27,28,29,30]. For instance, the copolymerization of polar monomers, particularly methyl acrylate (MA), with olefins confers novel properties on the resulting polymers: improved thermal stability, superior etch resistance, good adhesion due to the ester groups and a hydrophobic character derived from the C–C backbone [31,32,33]. Although recent progress in polymerization based on palladium catalysts allows the efficient copolymerization of polar and nonpolar monomers, the catalyst activity and second monomer insertion ratio remain quite low [34,35,36,37]. Subsequently, the design of suitable catalyst systems for efficient copolymerization of polar and non-polar monomers viz. acrylate and norbornene (NB) is still a great challenge [38,39,40,41,42,43].

**Figure 1:**
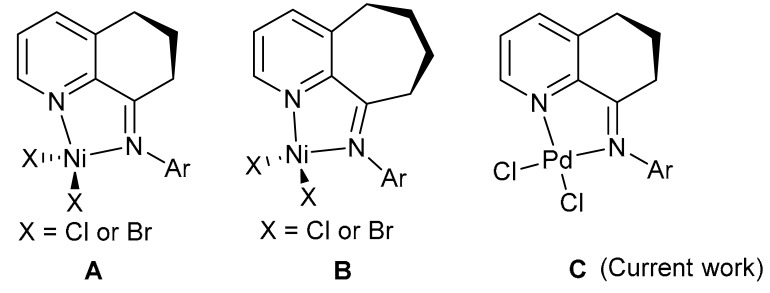
Geometry-constrained iminopyridylmetal halide complexes.

In view of the aforementioned perspectives and targeting higher efficiency catalysts, many newfangled ligand frameworks have been reported to provide subtle control over the palladium driven catalysis of either coupling reactions or polymerization. For instance; Nozaki et al. developed a highly efficient organic palladium catalyst framework for copolymerization of polar and nonpolar monomers [44]. Similarly, Chen et al. reported highly robust palladium(II) α-diimine catalysts for copolymerization of α-olefins with MA [45]. Besides, our group successfully established highly efficient nickel complexes comprising constrained cycloalkyl-fused pyridine ligands, viz *N*-(5,6,7-trihydroquinolin-8-ylidene)arylamine ligands [46] (Figure 1, **A**) and 9-arylimino-5,6,7,8-tetrahydro-cyclohepta[*b*]pyridine [47] (Figure 1, **B**). Meanwhile, recently highly efficient palladium complexes ligated with the same *N*-(5,6,7-trihydroquinolin-8-ylidene)arylamine ligands were reported for Heck and Suzuki coupling reactions [48]. Owing to these encouraging result, we were interested in extending the scope of *N*-(5,6,7-trihydroquinolin-8-ylidene)arylamine ligand-containing palladium complexes (Figure 1, **C**). In this research, we have prepared a pair of new palladium complexes **Pd4** and **Pd5** along with recently reported **Pd1**–**Pd3** complexes. Owing to the simplicity and ease of preparation of the title palladium complexes, we have employed them in MA polymerization and copolymerization of MA with NB under mild conditions. The resultant PMA has high molecular weight, up to 6282 kg·mol^−1^, and gave 98.4% conversion. The incorporation of NB was improved up to 18%. Additionally **Pd4** and **Pd5** were also used in the coupling of haloarenes with styrene substrates. Full characterization of new complexes and structural features of PMA and PM-co-NB polymer are reported.

## 2. Results and Discussion

### 2.1. Synthesis and Characterization of Palladium Complexes

**Scheme 1:**
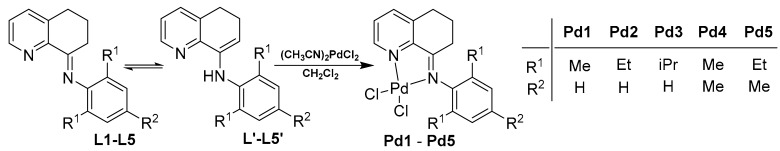
Synthesis of palladium complexes **Pd1**–**Pd5**.

In order to explore the catalytic potential of palladium complexes, a series of ligands **L1**–**L5** and their corresponding palladium complexes **Pd1**–**Pd5** have been prepared. Complexes **Pd1**–**Pd3** have already been reported as highly active catalysts for Heck and Suzuki coupling reactions [48]. On the basis of these encouraging results, we extended the scope of the reported **Pd1**–**Pd3** complexes and additionally synthesized the new compounds **Pd4** and **Pd5** for homopolymerization of MA and copolymerization of MA and NB. Additionally **Pd4** and **Pd5** were also employed in Heck coupling reactions. Ligands **L4** and **L5** and their complexes **Pd4** (51%) and **Pd5** (89%) have been prepared according to the same procedure reported in our previous work [46] as shown in Scheme 1. The new synthesized complexes were fully characterized by ^1^H-, ^13^C-NMR and FTIR spectroscopies as well as elemental analysis. All the distinctive peaks for proton and carbon atoms in the ^1^H- and ^13^C-NMR are consistent with the proposed structures of the palladium complexes. Moreover, the *V*_C=N_ stretching vibration absorption underwent a blue shift at around 1577 cm^−1^ in the FT-IR spectra of the palladium complexes, which is consistent with the reported data [49,50]. The palladium complexes are highly stable in the solid state as well as for several months in their solutions in haloalkanes (dichloromethane and chloroform), dimethylacetamide (DMA), dimethylformamide (DMF), toluene and acetonitrile. In addition, the molecular structures of both newly synthesized palladium complexes **Pd4** and **Pd5** have been determined by single crystal X-ray diffraction studies.

### 2.2. Single Crystal X-ray Diffraction Study

**Figure 2:**
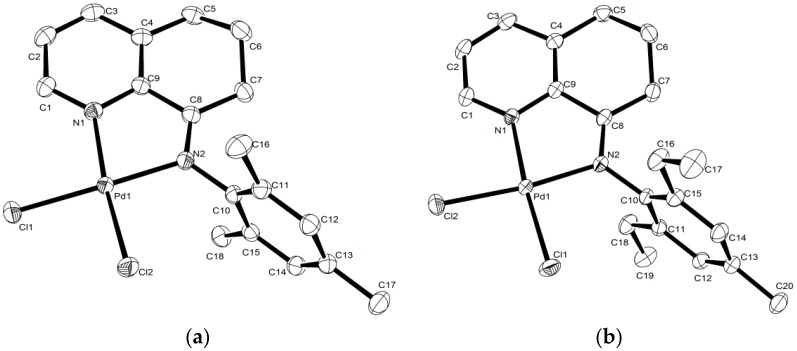
ORTEP diagrams of **Pd4** (**a**) and **Pd5** (**b**). Thermal ellipsoids set at the 30% probability level. Hydrogen atoms are omitted for clarity.

The crystals of complexes **Pd4** and **Pd5** were grown by layering diethyl ether on their dichloromethane solutions. Both complexes **Pd4** and **Pd5** displayed a similar molecular structure that adopts a distorted square planar geometry around the palladium metal center and the square plane is comprised of two nitrogen atoms from the ligand as forming a chelate ring with Pd metal center and two chloride atoms. The structural features correspond to classical *N*,*N*-bidentate palladium complexes [51,52,53,54,55,56]. The molecular structures of **Pd4** and **Pd5** are shown in Figure 2 and the selected bond lengths and bond angles are listed in Table S1 (ESI). The N2–C8 bond length shows double bonding features characteristic of imino groups: 1.292(3) Å in **Pd4** and 1.287(3) Å in **Pd5**, whilst the C8–C7 bond length characteristically revealed single bonding features: 1.494(3) Å (**Pd4**) and 1.497(3) Å (**Pd5**) contrary to the ligand which shows single as well as double bonding features [46]. The bite angle N(2)–Pd(1)–N(1): 80.31(7) for **Pd4** and 80.37(7) for **Pd5** was observed to be smaller than 90° which is accompanied by opening of the other three angles attributed to the non-aromatic six membered framework. In addition, the C2 atoms in the non-aromatic six membered framework fused with the pyridine ring are constrained from planarity, possibly due to minimization of the angle ring strain. The N_imine_ aryl ring is inclined nearly perpendicular with respect to the plane of the chelate ring N1–C9–C8–N2–Pd1 in **Pd4** (87.98°), while, slightly less twisting of the N_imine_ aryl ring is observed in case of **Pd5** (80.30°). Both bond lengths and bond angles are in good agreement with previously reported analogy palladium complexes [57].

### 2.3. Polymerization of MA and Copolymerization of MA/NB

**Figure 3:**
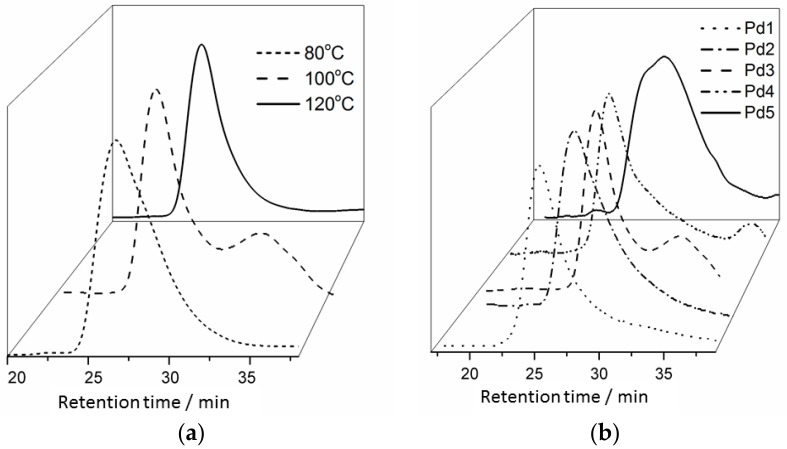
GPC traces of PMA produced by complex **Pd3** at different polymerization temperature (**a**) and by different complexes **Pd1**–**Pd5** (**b**).

To establish the optimal conditions for polymerization using a catalyst system composed of **Pd3** and (C_6_F_5_)_4_BC_6_H_5_NH(CH_3_)_2_, reaction temperature and run time were systematically investigated; the results are summarized in Table 1. Generally, N_2_PdCl_2_ type palladium complexes are sensitive to the temperature and easily dissociate at elevated temperature, subsequently affecting the catalytic activity of polymerization, hence homopolymerization of MA was conducted at different temperatures to explore the best catalyst activity. It was observed that when the temperature was increased from 80 °C to 120 °C, the highest conversion (95%) was obtained at 100 °C. In contrast, in polymerization either at lower or higher temperatures than 100 °C, **Pd3** exhibited lower activities, illustrating that partial deactivation started at elevated temperature. From the perspective of polymer microstructure, the raising temperature also causes in increase of the molecular weight of the polymers, consistent with the literature [58] and even higher than the corresponding polymerization data catalyzed by other late transition metals viz. Fe(II) and Co(II)pyridyl bis(imine)/MAO α-diimine Ni(II)/MAO systems [59]. Besides, the obtained PMAs show a broad bimodal molecular weight distribution with a high molecular weight tail at 100 °C as shown in the GPC traces in Figure 3.

To check the efficiency of the catalyst regarding the reaction time, the homopolymerization of MA has been performed for different time durations such as 1, 2, 3 and 4 h: conversion was increased from 24.2 to 95.0% as the reaction duration was prolonged from 1 to 4 h (Runs 2, 4, 5 and 6, Table 1). Interestingly, the molecular weight of the polymers increased regularly with the prolonged reaction time; a maximum molecular weight of 2252 kg·mol^−1^ was observed after 4 h reaction run time (Run 2, Table 1).

Under the optimized polymerization conditions, the catalytic behavior of additional complexes (**Pd1**, **Pd2**, **Pd4** and **Pd5**) was also examined. The order of catalytic efficiency observed was: **Pd5** > **Pd4** > **Pd3** > **Pd2** > **Pd1**. By examining the data in Table 1, complexes **Pd5** and **Pd4** showed improved conversion (Runs 9 and 10, Table 1) when compared with their analogous complexes **Pd2** and **Pd1,** respectively (Runs 7 and 8 Table 1). Likewise **Pd5** showed slightly higher conversion of MA as compared to **Pd4** and a similar correlation was observed by comparing the complexes **Pd3** with **Pd2** or **Pd1**. Contrary to Wu et al.’s observations [35,36], sterically hindered **Pd5** gave improved conversion, so it seemed a ligand effect was involved during the initial insertion of the MA monomer. Considering the molecular weight of the obtained polymers (Figure 3), **Pd5** gave the highest molecular weight, up to 6282 kg∙mol^−1^. However, **Pd1**, **Pd2**, **Pd4** and **Pd5** showed narrow polydispersity (M_w_/M_n_ = 3.21–3.98) while **Pd3** exhibited a broad polydispersity of 11.5. 

In order to verify either a free radical or coordination mechanism, MA polymerization was performed in the absence of ambient light under optimized reaction conditions (Run 11, Table 1). Polymerization was totally hindered in the absence of ambient light, confirming the free radical mechanism of our catalytic system. According to the mechanistic studies of Albéniz et al., free radicals were produced by light induced-homolytic cleavage of the Pd–C bond after the incorporation of one MA unit into the Pd–C_6_F_5_ bond of **1** [60]. A similar mechanistic pathway was also proposed by Ye et al. [35]. Although at this stage less experimental evidence is available, we reason that radicals were similarly produced from the light induced-decomposition of the palladium complex intermediate after initial incorporation of MA monomer into the palladium active species. Herein the palladium active specie could be formed only after the addition of (C_6_F_5_)_4_BC_6_H_5_NH(CH_3_)_2_ initiator followed by insertion of one MA monomer. In fact no polymerization was observed in the absence of initiator. Furthermore, ^13^C-NMR measurements of representative samples (Run 2, Table 1) indicated that the obtained PMAs are atactic. Similar results were also observed by Sen et al. and established a radical mechanism for such type of PMAs [61,62].

Polyolefins functionalized with polar and non-polar monomers remain an area of great interest for providing hybrid polymeric properties which afford useful polymeric materials in industry as well as academia. However, it remains a great challenge to design suitable catalyst systems for efficient copolymerization of MA with NB [34,39,40,41,42,43,59]. In this essence, we employed the **Pd3**/(C_6_F_5_)_4_BC_6_H_5_NH(CH_3_)_2_ catalytic system for copolymerization of MA with NB under the aforementioned optimum conditions for homopolymerization of MA (Run 2, Table 1). Various feed ratios of MA/NB (9:1–5:5) were used and results are tabulated in Table 2. 

**Figure 4:**
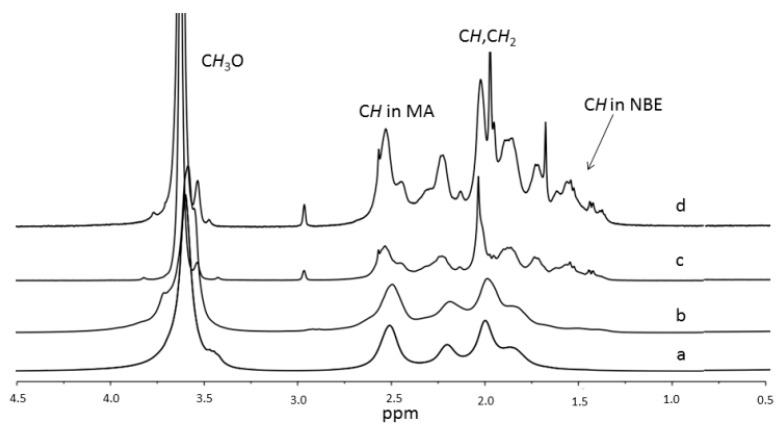
^1^H-NMR spectra of polymer: PMA (**a**), MA-*co*-NB originated from copolymerization with feed ratios of MA: NB = 9:1 (**b**), MA:NB = 7:3 (**c**), MA:NB = 5:5 (**d**).

**Figure 5:**
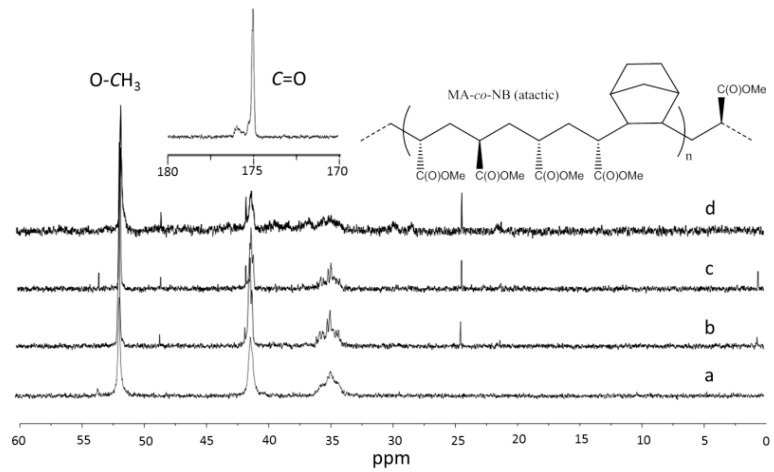
^13^C-NMR spectra of polymer: PMA (**a**), MA-*co*-NB originated from copolymerization with feed ratios of MA:NB = 9:1 (**b**), MA:NB = 7:3 (**c**), MA:NB = 5:5 (**d**).

It was observed that the incorporation of NB monomer gradually increased with an increase of feed ratio and a maximum 18% conversion was observed at a 5:5 feed ratio. The homopolymerization of NB did not give polymer using same **Pd3**/(C_6_F_5_)_4_BC_6_H_5_NH(CH_3_)_2_ catalytic system under similar reaction conditions. Conversely, 18% NB was incorporated during copolymerization of MA with NB, which assured the copolymerization was happened instead of simple physical mixing of both polymers. Further confirmation of copolymer formation was obtained from the by ^1^H- and ^13^C-NMR spectrum (vide infra, Figure 4 and Figure 5) [37,38]. As mentioned in Table 2, increasing the MA/NB feed ratio caused a substantial decrease in molecular weight of the obtained MA/NB copolymer from 2252 kg·mol^−1^ to 152 kg·mol^−1^. The molecular structure of PMA and MA-*co*-NB polymer was characterized by ^1^H- and ^13^C-NMR measurements. According to the ^1^H-NMR spectrum the methoxy peak appeared at 3.67 ppm and the methine and methylene protons of acrylate units as well as norbornene units appeared as broad resonances between 0.9–2.5 ppm illustrating the copolymerization was successful. The methylene protons for the acrylate units appeared around 1.98 ppm and overlapped with the methine protons from the norbornene as well as MA units. Furthermore, an intense methoxy group peak showed the MA enriched polymer was formed. All the assigned peaks are in agreement with the literature [61,63,64]. Moreover, as expected, no vibration for the *C=C* bond appeared at 1680–1620 cm^−1^ in FT IR spectrum indicating the absence of ring opening polymerization of NB [31].

Similarly, for comparison ^13^C-NMR spectra of PMA and MA-*co*-NB copolymer with different feed ratios (9/1, 7/3 and 5/5) are compiled together in Figure 5 and interpreted according to the literature [65]. The peaks for the carbonyl (–*C*(O)OMe), methoxy (–O*C*H_3_), methine (–*C*H) and methylene (–*C*H_2_–) carbons of PMA appear at 175.0 ppm, 51.8 ppm, 41.8 ppm and 35.0 ppm, respectively. On the other hand, in spectra (b) and (c), the methine and methylene carbon peaks became slightly less intense as well as broader. A clearer difference was observed in spectrum (c), where even the methylene carbon peaks of both monomer units emerge in the range of 34.0–44.1 ppm. Furthermore, the methoxy (–O*C*H_3_), methine (–*C*H) and methylene (–*C*H_2_–) signals in spectrum (b), (c) and (d) were slightly shifted toward higher field as compared to spectrum (a), verifying that the copolymerization had happened. A similar observation was reported by Wu and his co-workers [35,36]: NB are highly non-polar units which act as inert diluent that would be responsible for slightly shifting the chemical shift of MA segments to higher field. Such a shifting of peaks would not possible in a physic mixture of both polymers because no specific interaction between MA and NB monomers is possible. Moreover, some additional peaks was observed at 25.1 ppm, 30.2 ppm and 48.3 ppm which was assigned to the MA and NB sequence and the appearance of the peak intensity indicated a random sequence of co-monomers; such a sequence of MA and NB in polymer chains mostly occurs by a radical polymerization mechanism in agreement with the literature [34,39,40,41,42,43,59]. Though, a precise mechanism is unknown, like MA homopolymerization, MA free radicals are produced from the palladium intermediates, followed by propagation of MA-*co*-NB polymer chain by reacting with MA as well as NB monomers.

### 2.4. Heck Coupling Reaction

Generally the catalytic efficiency for Heck coupling reaction is highly influenced by the reaction conditions namely inorganic base, organic solvent, temperature and reaction time. In this context, firstly, the reaction conditions were optimized. The optimized reaction conditions were investigated by employing 0.002 mol% loading of **Pd5** complex for Heck coupling of classical substrates; *p*-bromo-toluene with 1.2 equivalents of styrene and explored the effect of base, temperature, solvent and reaction duration. The data are compiled in Table 3.

On careful inspection of the data, the best conversion was found when the coupling was performed in dimethylacetamide at 150 °C in the presence of K_2_CO_3_ for 12 h, which resulted in 98% conversion (Run 13, Table 3). Other bases including Na_2_CO_3_, NaHCO_3_, NaOAc and NaOH were also tested but showed lower efficiency (Runs 2–5, Table 3). In considering the crucial rule of the solvent in Heck coupling reactions, various polar and non-polar solvents were employed: the polar aprotic solvent *N*,*N*-dimethylformamide (DMF) exhibited similar activity as DMA and the coupling product could be obtained in 92% yield after 8 h reaction run time (Run 6, Table 3). An the other hand, due to lower boiling points of the solvents toluene and acetonitrile, the reaction was conducted at 100 °C and 60 °C respectively, but no conversion was observed (Runs 6–8, Table 3). In order to distinguish the effect of solvent or/and temperature, the coupling reaction was performed at lower temperature (60 °C and 100 °C) using the optimal solvent (DMA), and still no conversion was obtained, illustrating the dynamic role of temperature in the coupling reactions (Runs 9 and 10, Table 3). On the inspection of the reaction run time, surprisingly, slightly less conversion (93%) was observed after 8 h reaction run time (Run 1, Table 3). Less reaction time, namely 2 and 4 h, gave less conversion (Runs 12 and 13, Table 3), while 12 h reaction gave slightly more conversion, but considering good the ToF values we used eight hour reaction time to explore the scope for further reaction substrates. 

Using the optimum reaction conditions as established for **Pd5**, **Pd4**, PdCl_2_ and Pd(OAc)_2_ were additionally employed to evaluate the catalytic efficiency for the coupling of *p*-bromotoluene and styrene. According to the results as mentioned in Table 3, **Pd4** like **Pd5** exhibited excellent conversion (93%) and high ToF value (Run 14, Table 3). In contrast, PdCl_2_ and Pd(OAc)_2_ showed only 22% and 41% conversion, respectively, albeit at higher catalyst loading, illustrating the role of the ligand in the catalytic efficiency (Runs 15 and 16, Table 3). Complexes **Pd4** and **Pd5** have additional methyl groups on the *para*-position, which showed a detrimental effect that led to a slightly lower conversion rate as compared to analogous complexes [48]. 

Furthermore, keeping the optimal conditions in mind, the scope of the catalytic system **Pd5** was further investigated in the coupling of styrene with a variety of haloarene substrates as listed in Table 4. High catalytic efficiency was achieved in the case of bromobenzene (Run 1, Table 4), but the efficiency abruptly dropped in the case of 2,4,6-trimethylbromobenzene (Run 2, Table 4), being attributed to the steric crowding of both *ortho*-methyl substituents. 

However, 1,4-dibromobenzene gave 43% conversion in the first coupling stage and but second coupling of styrene seemed more difficult as it gave only 21% conversion caused of the high steric hindrance (Run 3, Table 4). Meanwhile, introducing electron donating groups, namely –NH_2_ and –OMe at the *para*-position of bromobenzene gave slightly less conversion as compared to bromobenzene: 77% and 76%, respectively (Runs 4 and 5, Table 4). In comparison with the self-encapsulated Pd-diimine catalyst reported by Ye et al., **Pd5** is an efficient catalyst showing high values of ToF (4000 and 3847 h^−1^) [66]. In the case of 3-methoxybromobenzene, the catalytic efficiency was further decreased up to 53% (Run 6, Table 4) consistent with the literature [67]. On comparison with our earlier reported work [48], the current catalytic system exhibited similar efficiency *viz* electron withdrawing groups positively enhanced the catalytic efficiency and electron donating groups showed contrasting results. At the same time, the coupling reaction of highly inert 2-bromopyridine substrates were also investigated with styrene (Runs 7 and 8, Table 4) but lower activity was exhibited, possibly due to coordination of N_pyridine_ with Pd metal center leading to lower efficiency. This indicated that it is worthy to explore more efficient catalyst systems for the coupling of less reactive substrates, albeit a plethora of highly efficient catalysts are available. The structures of the isolated products were elucidated by ^1^H- and ^13^C-NMR spectroscopy and interpreted according to the literature [68,69,70].

## 3. Experimental Section

### 3.1. General Information

All the manipulations of air and/or moisture sensitive compounds were conducted under a nitrogen atmosphere by standard Schlenk techniques. All the reagents were purchased from Aldrich (Beijing, China). NMR spectra were recorded on a DMX 400 MHz instrument (Bruker, Karlsruhe, Germany) at ambient temperature with TMS as an internal standard. FTIR spectra were determined on a System 2000 FTIR spectrometer (Perkin-Elmer, Shanghai, China). Elemental analysis was performed by an HPMOD 1106 microanalyzer (Thermo Electron SPA, Beijing, China).

### 3.2. Synthesis of Ligands ***L1**–**L5***

Ligands **L1**–**L5** have been prepared according to the procedure as reported in our previous study [47].

### 3.3. Synthesis of Palladium Complexes

A similar procedure was used as described for the synthesis of **Pd1**–**Pd3** in our previous study [48]. A solution of (CH_3_CN)_2_PdCl_2_ (2 mmol) in CH_2_Cl2 (20 mL) was added with the corresponding ligand (2 mmol) at room temperature. After overnight stirring, the color of the reaction mixture turned from light yellow to dark yellow, then the complex was precipitated by adding an excess of diethyl ether, filtered and washed with diethyl ether (3 × 5 mL). Finally, the precipitate was dried under vacuum afforded a yellow microcrystalline powder in high yield.

*N-(5,6,7-Trihydroquinolin-8-ylidene)-2,4,6-trimethylphenyliminopalladium(II) dichloride* (**Pd4**): Yellow microcrystalline solid, 51% yield. ^1^H-NMR (CDCl_3_): 9.33 (d, *J* = 8.0 Hz, 1H); 7.92 (d, *J* = 8.0 Hz, 1H); 7.66 (t, *J* = 6.0 Hz, 1H); 6.91 (s, 2H), 3.03 (t, *J* = 8.0 Hz, 2 H); 2.45 (t, *J* = 4.0 Hz, 2H); 1.99 (t, *J* = 6.0 Hz, 2H); 1.67 (s, 9H). ^13^C-NMR (CDCl_3_): δ 178.8, 152.3, 149.5, 142.7, 140.4, 140.2, 140.1, 129.0, 128.5, 123.7, 31.7, 28.8, 28.1, 23.6, 21.8. FTIR (KBr, disk, cm^−1^): 3074, 2965, 2906, 2876, 1604, 1577, 1453, 1376, 1288, 1190, 1128, 1035, 956, 851, 789, 660. Anal. calcd for C_18_H_20_Cl_2_N_2_Pd (442): C, 48.95; H, 4.56; N, 6.34. Found: C, 49.35; H, 4.68; N, 6.36.

*N-(5,6,7-Trihydroquinolin-8-ylidene)-2,6-diethyl-4-methylphenyliminopalladium(II) dichloride* (**Pd5**): Yellow microcrystalline solid, 89% yield: ^1^H-NMR (CDCl_3_): 9.35 (d, *J* = 4.0 Hz, 1H); 7.95 (d, *J* = 8.0 Hz, 1H); 7.68 (t, *J* = 6.0 Hz, 1H); 6.69 (s, 1H), 3.03 (t, *J* = 6.0 Hz, 2 H); 2.84-2.74 (m, 2H); 2.52–2.43 (m, 4H); 1.79 (t, *J* = 6.0 Hz, 2H), 1.29 (t, *J* = 6.0 Hz, 6H). ^13^C-NMR (CDCl_3_): δ 178.4, 152.4, 150.0, 142.3, 140.4, 139.5, 137.9, 134.7, 129.1, 126.8, 31.3, 28.4, 24.6, 22.1, 21.6, 13.7. FTIR (KBr, disk, cm^−1^): 3073, 2967, 2928, 2871, 1604, 1579, 1456, 1375, 1286, 1188, 1128, 934, 854, 791, 668. Anal. calcd for C_18_H_20_Cl_2_N_2_Pd (442): C, 51.14; H, 5.15; N, 5.96. Found: C, 50.97; H, 5.11; N, 5.93.

### 3.4. Homopolymerization of MA

Typical procedure (Run 2, Table 1): A 50 mL oven-dried Schlenk flask was charged with **Pd3** complex (9.2 mg, 20 μmol) and (C_6_F_5_)_4_BC_6_H_5_NH(CH_3_)_2_ (0.017 mg, 24 μmol, 1.2 equiv.) under nitrogen, then MA (8.6 g, 100 mmol) was injected into the reaction mixture by syringe. The reaction was stirred for 4 h at 80 °C. After the required time of polymerization, methanol was added to quench the polymerization. After filtering the polymer was dried under vacuum in an oven for 12 h at 45 °C. 

### 3.5. Copolymerization of MA and NB

Typical procedure: A 50 mL oven-dried Schlenk flask was charged with **Pd3** complex (9.2 mg, 20 μmol) and (C_6_F_5_)_4_BC_6_H_5_NH(CH_3_)_2_ (0.017 mg, 24 μmol, 1.2 equiv.) under nitrogen. Then the required molar ratio of MA and NB were added into the reaction mixture by syringe. After 4 h reaction time at 100 °C, methanol was added to quench the polymerization. The polymer was obtained by filtration and dried under vacuum in an oven for 12 h at 45 °C.

### 3.6. Heck Reaction

In a typical procedure for Run 1 in Table 3, a 50 mL oven-dried Schlenk flask was charged with *p*-bromotoluene (342 mg, 2.0 mmol), styrene (254 mg, 2.4 mmol), anhydrous K_2_CO_3_ (304 mg, 2.2 mmol) and dimethylacetamide (DMA, 4.0 mL) under nitrogen. A 4 µmol **Pd5** solution in DMA solvent was added into reaction mixture using a syringe, and then the reaction tube was kept stirring at 150 °C for 8 h. After cooling to room temperature, the reaction mixture was diluted with water and EtOAc. The combined organic extract was washed with brine, dried over Na_2_SO_4_ and concentrated. The crude product was purified by flash chromatography on silica gel to afford the desired product 1-methyl-4-styrylbenzene.

### 3.7. X-ray Crystallographic Study

Single crystals of **Pd4** and **Pd5** suitable for X-ray diffraction analysis were obtained from their dichloromethane solution using diethyl ether at room temperature. X-ray diffraction study were conducted on a Saturn 724 + CCD diffractometer (Rigaku, Tokyo, Japan) with graphite-monochromated Mo-Kα radiation (λ = 0.71073 Å) at 173(2) K. Cell parameters were obtained by global refinement of the positions of all collected reflections. Intensities were corrected for Lorentz and polarization effects and empirical absorption. The structures were solved by direct methods and refined by full-matrix least squares on F^2^. All hydrogen atoms were placed in calculated positions. Structure solution and refinement were performed by using the SHELXL–97 package [71]. Details of the X-ray structure determinations and refinements are provided in Table S2.

## 4. Conclusions

Along with reported complexes **Pd1**–**Pd3**, a pair of new complexes **Pd4** and **Pd5** have been prepared and fully characterized by ^1^H-NMR, ^13^C-NMR and FT-IR spectroscopies as well as elemental analysis. The molecular structures of **Pd4** and **Pd5** in the solid state have also been determined by X-ray diffraction, showing slightly distorted square planar geometry around the palladium metal center. All complexes **Pd1**–**Pd5** displayed excellent catalytic activity, with as high as 98% conversion, for MA polymerization. From the perspective of PMA microstructure, a high molecular weight (6282 kg·mol^−1^) was achieved. Moreover, **Pd3** has good capability in copolymerization of MA with NB and exhibted up to 18% incorporation. Additionally **Pd4** and **Pd5** was employed for the coupling of halorenes with styrene and gave almost quantitative conversions. In summary, the reported complexes are easy to prepare and are highly efficient catalysts for different applications. Further research on modification of these palladium complexes by changing the spectator ligands is undergoing.

## Figures and Tables

**Table 1:** The catalytic properties of Pd complexes for MA homopolymerization ^a^.

Run	Pre-Catalyst	T (°C)	Time (h)	Yield (g)	Conversion (%) ^b^	M_w_^c^ (kg∙mol^−1^)	M_w_/M_n_^c^
1	**Pd3**	80	4	7.30	77.0	1498	2.33
2	**Pd3**	100	4	9.01	95.0	2252	11.5
3	**Pd3**	120	4	6.01	63.4	1091	1.74
4	**Pd3**	100	1	2.21	24.2	1534	3.54
5	**Pd3**	100	2	5.22	56.1	1734	2.39
6	**Pd3**	100	3	7.44	79.4	1830	3.46
7	**Pd1**	100	4	7.70	82.0	1936	3.21
8	**Pd2**	100	4	8.76	93.4	1163	3.79
9	**Pd4**	100	4	9.15	97.2	1596	3.98
10	**Pd5**	100	4	9.28	98.4	6282	3.73
11 ^d^	**Pd3**	100	4	-	-	-	-

^a^ Conditions: 10.0 μmol of Pd complexes activated with 1.2 eq (C_6_F_5_)_4_BC_6_H_5_NH(CH_3_)_2_; 10 mL MA. ^b^ Calculated by ^1^H-NMR. ^c^ Determined by GPC. ^d^ Polymerization in absence of light.

**Table 2:** Copolymerization of MA and NB by complexes **Pd3** ^a^.

Run ^a^	MA/NBE	Yield (g)	Conversion of MA (%) ^b^	NB/MA ^b^	M_w_^c^/kg∙mol^−1^	M_w_/M_n_^c^
1	1:0	9.01	95	-	2252	1.74
2	9:1	7.38	68	8	1699	2.69
3	7:3	5.21	49	16	202	5.74
4	5:5	3.49	30	18	152	4.59
5	0:1	-	-	-	-	-

^a^ General conditions: 10.0 μmol of **Pd3** activated with 1.2 eq (C_6_F_5_)_4_BC_6_H_5_NH(CH_3_)_2_ at 100 °C. ^b^ Calculated by ^1^H-NMR. ^c^ Determined by GPC.

**Table 3:**
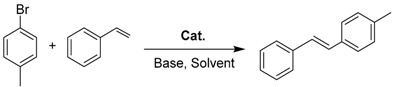
Optimization of reaction conditions using **Pd1** as catalyst ^a^.

Run	Catalyst	Base	T (°C)	Solvent	Time (h)	Conversion (%) ^b^	ToF ^c^
1	**Pd5**	K_2_CO_3_	150	DMA	8	93	5813
2	**Pd5**	Na_2_CO_3_	150	DMA	8	72	4500
3	**Pd5**	NaHCO_3_	150	DMA	8	74	4625
4	**Pd5**	NaOAc	150	DMA	8	82	5125
5	**Pd5**	NaOH	150	DMA	8	70	4375
6	**Pd5**	K_2_CO_3_	150	DMF	8	92	5750
7	**Pd5**	K_2_CO_3_	100	Toluene	8	-	-
8	**Pd5**	K_2_CO_3_	60	CH_3_CN	8	-	-
9	**Pd5**	K_2_CO_3_	100	DMA	8	-	-
10	**Pd5**	K_2_CO_3_	60	DMA	8	-	-
11	**Pd5**	K_2_CO_3_	150	DMA	2	86	21500
12	**Pd5**	K_2_CO_3_	150	DMA	4	88	11000
13	**Pd5**	K_2_CO_3_	150	DMA	12	98	4083
14	**Pd4**	K_2_CO_3_	150	DMA	08	93	5813
15 ^d^	PdCl_2_	K_2_CO_3_	150	DMA	08	22	28
16 ^d^	Pd(OAc)_2_	K_2_CO_3_	150	DMA	08	41	50

^a^ Conditions: 4 × 10^−5^ mmol **Pd5** as catalyst, 2.0 mmol *p*-bromotoluene, 2.4 mmol styrene, 2.2 mmol base, 4.0 mL solvent. ^b^ GC yield. ^c^ ToF = product/(catalyst × time), ^d^ Conditions: 2 × 10^−3^ mmol Pd, 2.2 mmol Na_2_CO_3_ as a base [56].

**Table 4:** Heck reaction of various aryl bromides and styrene using complex **Pd5** ^a^.

Run	Ar–X	Yield (%) ^b^	Conversion (%) ^c^	ToF ^d^
1	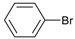	74	81	3905
2	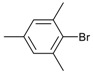	-	3.6	225
3	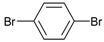	31 (15)	43 (21)	1938 (938)
4	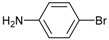	64	77	4000
5	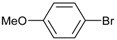	65	76	3847
6	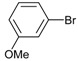	37	53	2312
7	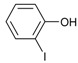	-	6	375
8	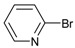	-	8	500

^a^ Conditions: 4 × 10^−5^ mmol Pd complex as the catalyst, 2.0 mmol *p*-bromotoluene, 2.4 mmol styrene, 2.2 mmol base, 4.0 mL DMA, 150 °C for 8 h. ^b^ Isolated yield. ^c^ GC yield. ^d^ ToF = product/(catalyst × time).

## Supplementary Materials

CCDC 1507785 and 1507784 contain the supplementary crystallographic data for **Pd4** and **Pd5**. These data can be obtained free of charge from The Cambridge Crystallographic Data Centre via www.ccdc.cam.ac.uk/data_request/cif. Table S1: the selected bond lengths and bond angles of **Pd4** and **Pd5**. Table S2: Crystal data and structure refinement for complexes **Pd4** and **Pd5**. Supplementary materials can be accessed at: http://www.mdpi.com/1420-3049/21/12/1686/s1.
