# Supplementary material for: Geometry Constrained N-(5,6,7-Trihydroquinolin-8-ylidene)arylaminopalladium Dichloride Complexes: Catalytic Behavior toward Methyl Acrylate (MA), Methyl Acrylate-co-Norbornene (MA-co-NB) Polymerization and Heck Coupling
Source: Molecules. 2016 Dec 7;21(12):1686. doi: 10.3390/molecules21121686 (PMC6273177; doi:10.3390/molecules21121686)
Supplement: Supplementary file 1 [file molecules-21-01686-s001.pdf]

Supplementary Materials: Geometry Constrained *N*-(5,6,7-Trihydroquinolin-8-ylidene)arylamino palladium Dichlorides Complexes: Catalytic Behavior toward MA, MA-*co*-NB Polymerization and Heck Coupling

Yanning Zeng, Qaiser Mahmood, Tongling Liang and Wen-Hua Sun

Table S1. Selected bond lengths (Å) and angles (°) for complexes **Pd4** and **Pd5**.

**Pd4**		**Pd5**	
Bond lengths (Å)			
Pd(1)-N(2)	2.0209(17)	Pd(1)-N(2)	2.0354(17)
Pd(1)-N(1)	2.0254(18)	Pd(1)-N(1)	2.0253(18)
Pd(1)-Cl(2)	2.2734(11)	Pd(1)-Cl(2)	2.2973(8)
Pd(1)-Cl(1)	2.2983(9)	Pd(1)-Cl(1)	2.2728(11)
N(1)-C(1)	1.330(2)	N(1)-C(1)	1.331(3)
C(7)-C(8)	1.494(3)	C(7)-C(8)	1.497(3)
Bond angles (°)			
N(2)-Pd(1)-N(1)	80.31(7)	N(2)-Pd(1)-N(1)	80.37(7)
N(2)-Pd(1)-Cl(2)	94.26(5)	N(1)-Pd(1)-Cl(1)	174.96(5)
N(1)-Pd(1)-Cl(2)	172.03(5)	N(2)-Pd(1)-Cl(1)	94.63(6)
N(2)-Pd(1)-Cl(1)	171.81(5)	N(1)-Pd(1)-Cl(2)	94.28(6)
N(1)-Pd(1)-Cl(1)	95.33(6)	N(2)-Pd(1)-Cl(2)	174.05(5)
Cl(2)-Pd(1)-Cl(1)	90.75(3)	Cl(1)-Pd(1)-Cl(2)	90.69(4)
C(1)-N(1)-C(9)	119.13(17)	C(1)-N(1)-C(9)	119.16(17)
C(1)-N(1)-Pd(1)	127.64(14)	C(1)-N(1)-Pd(1)	127.93(15)
C(9)-N(1)-Pd(1)	113.19(13)	C(9)-N(1)-Pd(1)	112.90(13)
C(8)-N(2)-C(10)	120.44(16)	C(8)-N(2)-C(10)	121.19(17)
C(8)-N(2)-Pd(1)	115.48(13)	C(8)-N(2)-Pd(1)	114.78(13)
C(10)-N(2)-Pd(1)	123.76(12)	C(10)-N(2)-Pd(1)	124.03(13)

Table S2. Crystal data and structure refinement for complexes **Pd4** and **Pd5**.

	**Pd4**	**Pd5**
Crystal color	Yellow	Yellow
Empirical formula	$C_{18}H_{20}Cl_2N_2Pd$	$C_{20}H_{24}Cl_2N_2Pd$
Formula weight	441.66	469.73
Temperature/K	173 (2)	173 (2)
Wavelength/Å	0.71073	0.71073
Crystal system	Monoclinic	Triclinic
Space group	P21/a	P-1
a/Å	7.4548(15)	8.3628(17)
b/Å	30.651(6)	8.5040(17)
c/Å	8.2911(17)	15.861(3)
Beta/°	112.43(3)	74.93(3)
Volume/$Å^3$	1751.2(6)	966.4(3)
Z	4	2
D_{calcd}/(g·cm^{-3})	1.675	1.614
μ/mm^{-1}	1.365	1.242
F(000)	888	476
Crystal size/mm	0.39 × 0.27 × 0.07	0.46 × 0.20 × 0.11
θ range (°)	2.66–27.49	2.79–24.99
Limiting indices	$-9 \le h \le 9$<br>$-39 \le k \le 39$<br>$-10 \le l \le 10$	$-9 \le h \le 9$<br>$-10 \le k \le 10$<br>$-18 \le l \le 18$
No. of rflns collected	18,759	10,319
No. unique rflns	3989	3351
R(int)	0.0296	0.0234
No. of params	211	229
Completeness to θ	99.1%	98.9%
Goodness of fit on F^2	0.986	1.039
Final *R* indices [$I > 2\sum(I)$]	$R1 = 0.0278$<br>$wR2 = 0.0726$	$R1 = 0.0197$<br>$wR2 = 0.1208$
R indices (all data)	$R1 = 0.0283$<br>$wR2 = 0.0732$	$R1 = 0.0201$<br>wR2 = 0.0555
Largest diff. peak, and hole/(e·$Å^{-3}$)	1.206 and −0.706	0.379 and −0.458
